# Identification, Classification and Screening for γ-Amino-butyric Acid Production in Lactic Acid Bacteria from Cambodian Fermented Foods

**DOI:** 10.3390/biom9120768

**Published:** 2019-11-22

**Authors:** Dalin Ly, Sigrid Mayrhofer, I. B. Agung Yogeswara, Thu-Ha Nguyen, Konrad J. Domig

**Affiliations:** 1 Department of Food Science and Technology, BOKU-University of Natural Resources and Life Sciences Vienna, Muthgasse 18, A-1190 Vienna, Austria; sigrid.mayrhofer@boku.ac.at (S.M.); ida.agung-yogeswara@boku.ac.at (I.B.A.Y.); thu-ha.nguyen@boku.ac.at (T.-H.N.); konrad.domig@boku.ac.at (K.J.D.); 2 Department of Food Biotechnology, Faculty of Agro-Industry, Royal University of Agriculture, Dangkor District, P.O. Box: 2696, Phnom Penh 12400, Cambodia; 3 Department of Nutrition, Universitas Dhyana Pura, Bali 80361, Indonesia

**Keywords:** lactic acid bacteria, fermented foods, 16S rDNA sequencing, MALDI-TOF MS, (GTG)_5_ rep-PCR fingerprinting, GABA, Cambodia

## Abstract

Screening for various types of lactic acid bacteria (LAB) that form the biological agent γ-amino-butyric acid (GABA) is important to produce different kinds of GABA-containing fermented foods. So far, no GABA-producing LAB have been reported from Cambodian fermented foods. Most small-scale fermentations and even some industrial processes in this country still rely on indigenous LAB. The application of GABA-producing autochthonous starters would allow the production of Cambodian fermented foods with an additional nutritional value that meet the population’s dietary habits and that are also more attractive for the international food market. Matrix-assisted laser desorption/ionizing time-of-flight mass spectrometry (MALDI-TOF MS) and partial 16S rDNA sequencing were used to identify 68 LAB isolates from Cambodian fermented foods. These isolates were classified and grouped with (GTG)_5_ rep-PCR, resulting in 50 strains. Subsequently, all strains were investigated for their ability to produce GABA by thin layer chromatography. GABA-positive strains were further analyzed by the GABase assay. Of the six GABA-positive LAB strains—one *Lactobacillus futsaii*, two *Lactobacillus namurensis*, and three *Lactobacillus plantarum* strains—two *Lactobacillus plantarum* strains produced high amounts of GABA (20.34 mM, 16.47 mM). These strains should be further investigated for their potential application as GABA-producing starter cultures in the food applications.

## 1. Introduction

Fermented foods are widely consumed in Cambodia. In particular, fermented fish are eaten in almost every meal in most parts of the country and fermented fruits and vegetables are popular among females who eat them as a snack. Most of these food products are produced at household and small-scale levels. The majority of small-scale fermentations and even some industrial processes are still done as natural processes involving lactic acid bacteria (LAB) that are indigenously present in raw materials and the production environment. This represents a low-cost and reliable preservation technique [1]. Although LAB are generally recognized as safe (GRAS) by the US Food and Drug Administration (FDA) and partly have the qualified presumption of safety (QPS) status provided by the European Food Safety Authority (EFSA) [2,3], the growth of this group is completely uncontrolled and unpredictable, resulting in less uniform sensory characteristics and compositions [4]. Tailored commercial starter cultures would guarantee obtaining products with constant hygienic and organoleptic qualities in a shorter time and might also improve the stability and shelf life [5]. Since there is no commercial production of starter cultures for Cambodian products, universal cultures from other sources have to be used. Such cultures, however, are tailored to the needs of other markets and are not typical for Cambodian foods [4]. Moreover, many small-scale manufacturers are unwilling to accept changes and to modify fermentation processes [1]. Using autochthonous starter cultures, which can effectively preserve typical characteristics of fermented products, would be better accepted by producers [5]. A recent trend in food preservation is the use of safe starter cultures that show additional positive functions apart from technological and possible antimicrobial properties as well as stress resistance [4,5]. Such starter cultures would allow the production of Cambodian fermented foods with an additional nutritional value that meet the population’s dietary habits and that are also more attractive for the international food market.

There is currently considerable research and industrial interest in the potential biological activity of LAB, either as probiotics themselves or as producers of bioactive agents [6]. One of these safe and eco-friendly bioactive agents is γ-aminobutyric acid (GABA), which is a non-protein amino acid [7]. It is formed by the decarboxylation of L-glutamic acid in a reaction catalysed by the enzyme glutamate decarboxylase (GAD) [8]. Although GABA is found in many plants and animals, its content is generally low [9]. The application of concentrated GABA is wide and versatile, as this component has many physiological functions such as the induction of hypotension, neurotransmission, diuretic and sedative effects as well as the stimulation of immune cells [6]. Consequently, GABA is used in pharmaceuticals and functional/fermented foods as active component [10]. 

Because of the increasing commercial demand, there have been many attempts for synthesizing GABA chemically or biologically [10]. Since biological methods using microorganisms are more promising [11,12], many GABA products are obtained by fermentation [8]. Ongoing efforts in the molecular evolutions of GADs offer new prospects for effective GABA biosynthesis [13]. Next to LAB, other microorganisms have been reported for GABA production, including bacteria, fungi and yeasts [12,14]. However, LAB are the most interesting and practical group for this fermentation as they can produce high levels of GABA due to a high cellular GAD activity [10]. Although many GABA-producing LAB strains have already been isolated and identified, further research on the isolation and characterization of LAB is needed. Screening for new strains of LAB that can produce GABA still attracts attention because LAB with different physiological characteristics show potential for use as starters in the food industry to produce GABA-containing fermented foods with different acid and flavor profiles [14]. 

So far, no GABA-producing LAB starter cultures have been reported from Cambodian fermented foods. Thus, the objective of the present study was the identification of LAB from various Cambodian fermented fishery and vegetable products followed by an investigation of their ability to produce GABA. Hence, the first stage is to establish the precise identity of an isolate at genus and species level [4]. Currently, molecular techniques provide an important contribution to the identification and classification of microorganisms [15,16]. The most commonly used target for bacterial identification is the 16S rDNA (16S rRNA gene). Sequencing this gene is considered to be the ‘gold standard’ for solving bacterial phylogeny and taxonomy issues in different contexts [17]. Additionally, proteomic analysis based on protein profiling using matrix-assisted laser desorption/ionizing time-of-flight mass spectrometry (MALDI-TOF MS) has been recognized as a tool for microbial identification with high sensitivity and throughput [18,19]. To exclude possible duplicates from further analyses, a discrimination at strain level was performed using repetitive element palindromic (rep)-PCR. Subsequently, well-identified and characterized strains were screened for GABA production by thin layer chromatography (TLC), and the level of GABA produced was determined by GABase assay. 

## 2. Materials and Methods

### 2.1. Fermented Food Samples and Sampling

Eight types of naturally prepared Cambodian fermented foods (mainly fermented fish and vegetables) were randomly purchased from wet markets (Chamkadaung, Oreusey, Thmey, Chas, Phumreusey, and Limcheanghak) in Phnom Penh, the capital city of Cambodia. These products originated from various provinces in the country. The samples included fish paste (*prahok*; *n* = 1), fermented fish (*paork chav*; *n* = 3 and *mam trey*; *n* = 1), salted fish (*trey proheum*; *n* = 2), shrimp paste (*kapi*; *n* = 1), fermented papaya (*mam lahong*; *n* = 2), fermented mustard (*spey chrouk*; *n* = 2) and fermented tiny freshwater shrimp (*paork kampeus*; *n* = 3). The information about each fermented product is provided in Table 1. After purchasing fermented foods from the wet markets in Phnom Penh, samples were immediately packed into hygienic plastic boxes. The samples were taken to the Food Microbiology and Hygiene Laboratory of the Department of Food Science and Technology at BOKU in Vienna, Austria, and kept in their original containers at 4 °C until analysis. Cambodian fermented foods have mostly no shelf-life indicated and these foods are usually stored until completely consumed [1]. Generally, fermented fish can be stored for a few months up to a year and fermented vegetables are still fine up to two or three weeks if they are stored at 4 °C. To cover the purpose of the project, all analyses were carried out as soon as possible within the usual shelf life of the products (e.g., three months for fermented fishery products and two weeks for fermented vegetables after purchasing).

### 2.2. Growth Conditions and LAB Isolation

Ten grams (10 g) of each sample were aseptically taken, transferred into a stomacher bag, and homogenized (Stomacher 400 Circulator, Seward Ltd, Worthing, UK) with 90 mL buffered peptone water for 45 s at 230 rpm. Appropriate decimal dilutions of the samples were prepared using the same medium. From each dilution, 0.1 mL were inoculated on DeMan Rogosa Sharpe (MRS) agar (Merck, Darmstadt, Germany) by the spread plate method. Inoculated plates were incubated at 30 °C for 72 h in an anaerobic chamber (80% N_2_, 10% CO_2_, 10% H_2_, Scholzen Technik, Kriens, Switzerland). Subsequently, colonies with different morphologies were selected and streaked onto MRS agar for purification. After three days of anaerobic incubation at 30 °C, pure isolates were Gram-stained. Only colonies with gram-positive cocci or rods were transferred into 3 mL MRS broth (Merck) and anaerobically incubated for 24 h at 30 °C. The incubated MRS broth of each isolate was mixed with glycerol (99.5%, Roth, Karlsruhe, Germany) to obtain a final concentration of 20% glycerol (*v*/*v*) and stored at −80 °C. 

### 2.3. LAB Identification by Partial 16S rDNA Sequencing and MALDI-TOF MS

#### 2.3.1. DNA Extraction and Identification of LAB by Partial 16S rDNA Sequencing

Before DNA extraction, LAB isolates were resuscitated in MRS broth at 30 °C for 48 h. Genomic DNA of the isolates was then extracted using the peqGOLD Bacterial DNA Mini Kit (PeqLab, Erlangen, Germany) according to the manufacturer’s instructions. Afterwards, the DNA preparations were stored at −20 °C until use. The extracted DNA was used as template for 16S rDNA sequencing. PCR amplifications were performed in a total volume of 25 µL containing 1 µL of DNA, respectively. Moreover, 1 µL each of the forward primer bak4 (5′-AGG AGG TCA TCC ARC CGCA-3′; 10 pmol/µL) and the reverse primer bak11w (5′-AGT TTG ATC MTG GCT CAG-3′; 10 pmol/µL), 2.5 µL of 10× PCR buffer (Dynazyme buffer 10×; Thermo Scientific, Waltham, MA, USA), 0.5 µL of deoxynucleoside triphosphate (dNTP) mix (10 nmol/µL of each dNTP; GE Healthcare, Buckinghamshire, UK), 0.5 µL of DNA polymerase (2 U/µL; Dynazyme II; Thermo Scientific), and 18.5 µL of sterile distilled water were added. The following PCR program was applied: an initial denaturation at 95 °C for 3 min, 30 cycles of denaturation at 95 °C for 30 s, annealing at 56 °C for 30 s, extension at 72 °C for 2 min and a final extension at 72 °C for 7 min. PCR was conducted in an Eppendorf Mastercycler (Eppendorf, Hamburg, Germany). The obtained PCR products were analysed with a DNA ladder (GeneRuler 100 bp DNA ladder extended) by electrophoresis on a 2% (*w*/*v*) agarose gel in 0.75 × TAE buffer at 80 V for 110 min, stained with GelRed Nucleic Acid Gel Stain (Biotium, Fremont, CA, USA), and visualized with an ultraviolet transilluminator (Bio-RAD, Hercules, CA, USA).

PCR products thereof were purified with the QIAquick PCR Purification Kit (Qiagen, Venlo, The Netherlands) and sent to commercial sequencing (Eurofins MWG Operon, Ebersberg, Germany). Upon receipt of the data, sequences were aligned to the National Center for Biotechnology Information (NCBI) database using the Basic Local Alignment Search Tool (BLAST) with the BLASTn program. An unknown isolate was generally assigned to a species in the database whose sequence had a nearest neighbour exhibiting the highest similarity score of ≥97%.

#### 2.3.2. Identification of LAB by MALDI-TOF MS 

The isolates were identified using MALDI-TOF MS with the Bruker Biotyper (Bruker Daltonics, Bremen, Germany). The identification was conducted by the “extended direct transfer” and the “formic acid extraction” procedure according to the manufacturer’s instruction. For the “extended direct transfer” technique, a single colony of each isolate was deposited directly on a steel MSP96 target plate and subsequently overlaid with 1 μL of 70% formic acid (Roth) and air-dried. For the “formic acid extraction” method, a single colony or several colonies were placed into an Eppendorf tube containing 300 μL deionized water and mixed thoroughly. Then, 900 μL of ethanol (99.7%, VWR Chemicals, Fontenay-sous-Bois, France) was added and mixed. After that, samples were centrifuged at 14,000 rpm for 2 min and the supernatant was decanted. To remove the residual ethanol, the centrifugation step was repeated under the same conditions. Subsequently, 10 μL (depending on the size of the pellet) of 70% formic acid was added together with 10 µL pure acetonitrile (99.9%, VWR Chemicals, Fontenay-sous-Bois, France) and mixed thoroughly. After a further centrifugation step for 2 min at 14,000 rpm, 1 μL of the supernatant was applied onto a steel MSP96 target plate and air-dried at ambient temperature. Next, samples on the MSP96 target plate from both procedures were overlaid with 1 μL of matrix solution (10 mg/mL of α-cyano-4-hydroxycinnamic acid (HCCA) in acetonitrile:water:trifluoroacetic acid, 50:47.5:2.5 [*v*/*v*/*v*]). After the matrix solution was air-dried at ambient temperature, the plate was immediately applied to the MALDI-TOF Biotyper chamber (Bruker Daltonics) for analysis. Measurements were taken using a Microflex LT bench-top mass spectrometer (Bruker Daltonics) controlled by the FlexControl software (version 3.4; Bruker Daltonics). Three independent experiments were conducted for each isolate. Mass spectra were processed using the Biotyper software (version 4.1; Bruker Daltonics) and the BioTyper database containing 8223 reference MALDI-TOF MS profiles. The reliability of identification by the MALDI Bruker Biotyper system was expressed in points. A log(score) of ≥2.00 [green color (+++)] indicated identification to the species level and a log(score) of ≥1.70 and <2.00 [yellow color (+)] indicated identification to the genus level, while a score value under 1.70 [red color (-)] means no significant similarity between the unknown profile and any reference profile. If the log(score) was <2.00 (+), the “formic acid extraction” procedure was applied. 

### 2.4. Fingerprinting and Typing of LAB by (GTG)_5_-PCR

Repetitive element palindromic (rep-PCR) using the (GTG)_5_ primer (5´-GTG GTG GTG GTG GTG-3´) was performed [20] with a few modifications. Briefly, 1 μL of DNA was pipetted into 24 μL of a PCR mixture containing 1 µL of the (GTG)_5_ primer (50 pmol/µL), 0.5 μL of dNTP mix (10 nmol/µL of each dNTP; GE Healthcare), 0.5 µL of DNA polymerase (2 U/µL; Dynazyme II; Thermo Scientific), 2.5 μL of 10× PCR buffer (100 nmol/µL Tris-HCl, 15 nmol/µL MgCl_2_, 150 nmol/µL KCl, 0.1% Triton X-100; pH 8.8, Thermo Scientific), and 19.5 µL sterile distilled water. The cycling program of the Eppendorf Mastercycler (Eppendorf, AG) consisted of an initial denaturation step at 94 °C for 7 min, 30 cycles of denaturation at 90 °C for 30 sec, annealing at 40 °C for 1 min, extension at 65 °C for 8 min and a final extension at 65 °C for 16 min. Obtained PCR products were separated on a 2% agarose gel and stained with GelRed Nucleic Acid Gel Stain.

The cluster analysis of the rep-PCR profiles was performed on similarity matrices, which were produced using the Dice’s coefficient [21] and subjected to the unweighted pair group method with arithmetic mean (UPGMA) clustering algorithm using the BioNumerics software version 7.6.1 (Applied-Maths, Saint-Martens-Latem, Belgium). A tolerance level of 1% and an optimization of 0.5% were chosen for creating the dendrogram.

### 2.5. Screening LAB Strains for GABA Production Using Thin Layer Chromatography (TLC)

All identified LAB strains were screened by TLC using the method described [22] with some modifications. Briefly, all strains were cultured in 4 mL MRS broth supplemented with 2% monosodium glutamate (MSG; Sigma-Aldrich, St. Louis, MO, USA) and anaerobically incubated at 30 °C for 2–3 days. After incubation, the culture broth was centrifuged at 8700 rpm at 4 °C for 5 min. One microliter of supernatant from each strain was spotted on a silica TLC plate (Aluminum Sheets Silica gel 60 F254, Merck). GABA separation by TLC was conducted using a solvent mixture (1-butanol:acetic acid:distilled water, 5:2:2 *v*/*v*/*v*). GABA spots were detected after spraying 0.5% (*w*/*v*) ninhydrin on the plates and heating at 105 °C for 5 min. GABA standard (Sigma-Aldrich) and MSG were used as control standards. This analysis was performed in triplicate. The retention factor (Rf), defined as the ratio of the distance traveled by the center of a spot to the distance traveled by the solvent front, was calculated. Cultures of strains showing the same Rf value as the GABA standard were selected for quantification by the GABase assay.

### 2.6. Quantification of GABA Production

The GABA concentration of GABA-producing strains was determined by a spectrophotometric GABase assay as described [23] with modifications. Briefly, the reaction mixture (190 µL) contained 140 µL of 100 mM K_4_P_2_O_7_ buffer (pH 8.6), 30 µL of 4 mM NADP+, 10 µL of GABase (1 unit/mL, Sigma-Aldrich), and 10 µL of the standard solution (GABA) or culture supernatant (see 2.5.). This mixture was dispensed into each well of a 96-well plate. Before adding 10 µL of 20 mM α-ketoglutarate, the initial absorbance was read at 340 nm in a Multiskan FC plate reader (Thermo Fisher Scientific). The final absorbance was read again after 60 min incubation at room temperature at the same wavelength. The difference of both A340 values due to the conversion of NADP+ to NADPH was used to calculate the GABA content in the sample. The equation of the GABA standard curve was A340 = 0.0341x − 0.0154 (R^2^ = 0.9995), where x is the GABA concentration of the sample in mM. This analysis was done in triplicate.

### 2.7. Statistical Analysis

All statistical analyses were conducted with Microsoft Excel 2016 (Microsoft, Redmond, WA, USA). The data were checked for plausibility and validity. Descriptive statistics were applied by calculating the mean and standard deviations (SD) of the test results.

## 3. Results and Discussion

### 3.1. LAB Identification by Partial 16S rDNA Sequencing and MALDI-TOF MS

#### 3.1.1. Identification of LAB by Partial 16S rDNA Sequencing

**Figure 1:**
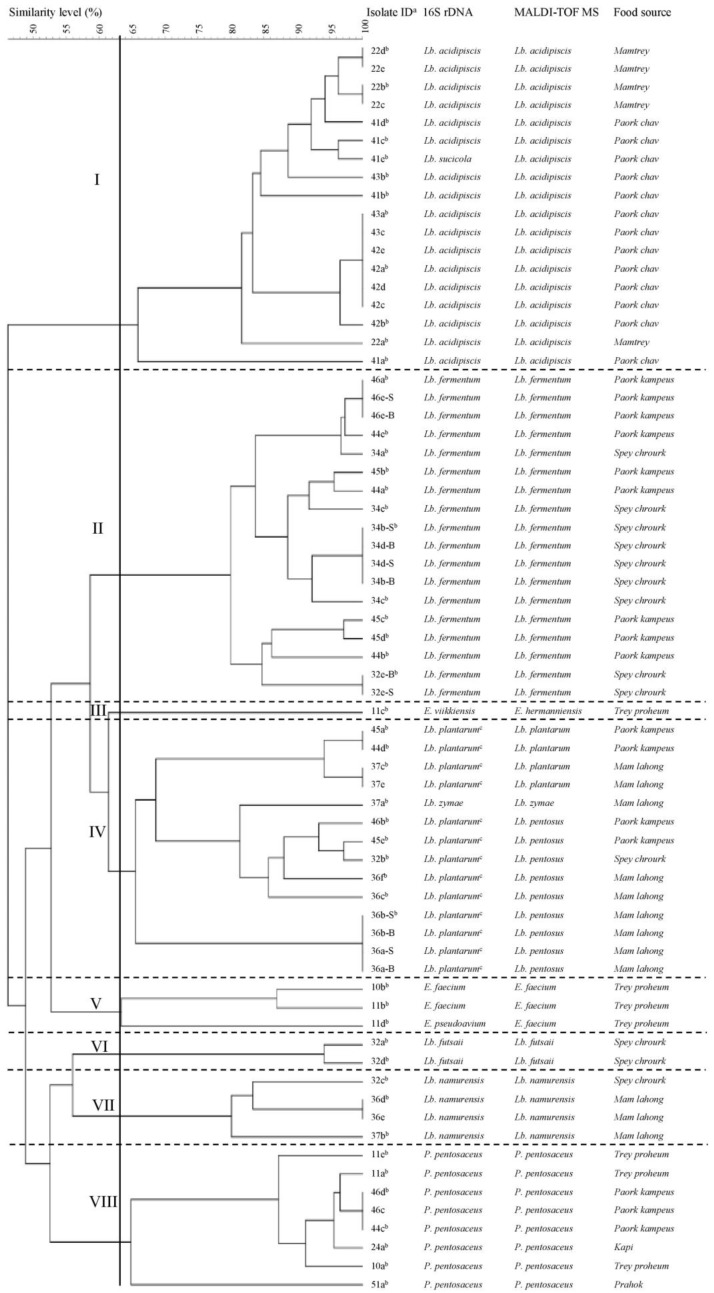
Dendrogram based on cluster analysis of rep-PCR (GTG)_5_ fingerprints obtained for LAB isolated from Cambodian fermented foods and identified at species level by partial 16S rDNA sequencing and MALDI-TOF MS (Bruker Biotyper). The dendrogram was constructed by the unweighted pair group method using arithmetic (UPGMA) mean with similarity levels expressed as percentage values of the Dice correlation coefficient. All isolates with a similarity of 100% and the same source were regarded as multiple isolates representing a single strain. ^a^ Identity. ^b^ Single strain used for GABA screening by TLC. ^c^ *Lb. plantarum*/*Lb. paraplantarum*/*Lb. pentosus* cannot be distinguished by partial 16S rDNA sequencing.

Isolating LAB from different types of Cambodian fermented foods, 96 isolates were gram-positive cocci (19 isolates) or rods (77 isolates). Of these, 68 isolates were confirmed as LAB, including 56 *Lactobacillus* isolates [*Lb. fermentum* (18), *Lb. acidipiscis* (17), *Lb. plantarum/paraplantarum/pentosus* (13), *Lb. namurensis* (four), *Lb. futsaii* (two), *Lb. zymae* (one), *Lb. sucicola* (one)], eight *Pediococcus* isolates [*P. pentosaceus* (eight)], and four *Enterococcus* isolates [*E. faecium* (two), *E. pseudoavium*/*E. avium* (one), *E. viikkiensis*/*E. durans*/*E. malodoratus*/*E. pseudoavium* (one)] (Figure 1). The remaining 28 isolates were bacilli, clostridia and staphylococci (data not shown). Our results strongly support previous findings, which stated that in some cases sequencing of the 16S rDNA has a limited discriminating and low phylogenetic power for several closely related lactobacilli and enterococcal species [24,25,26,27,28] due to substantial similarities of their 16S rDNA sequences [29,30,31,32]. Correspondingly, it was impossible to distinguish the three species *Lb. plantarum*, *Lb. paraplantarum* and *Lb. pentosus* in our study because their partial 16S rDNA sequences were highly similar (≥99%) (Table S1). The identification of enterococcal species is also challenging. *E. pseudoavium* and *E. avium* as well as *E. viikkiensis*, *E. durans*, *E. malodoratus*, and *E. pseudoavium* showed similarities of 96% and 99%, respectively (Table S1). Therefore, a few *Lactobacillus* spp. and *Enterococcus* spp. can in some cases only be correctly identified by combining several methods [33,34].

#### 3.1.2. Identification of LAB by MALDI-TOF MS

MALDI-TOF MS has been widely used for the rapid identification and taxonomic characterization of *Lactobacillus* spp. [15,30,34,35,36,37,38,39,40,41,42], *Enterococcus* spp. [33], and *Pediococcus* spp. [43] of different origin. The 68 LAB isolates of this study were firstly investigated using the “extended direct transfer” procedure. Hence, 38 isolates (55.9%) were identified at species level (score ≥2.00). For the remaining 30 isolates (44.1%), which had a primary score of <2.00, the “formic acid extraction” procedure was applied. Hence, it is known that the simple and rapid “extended direct transfer” protocol is inferior in accuracy because of insufficient cell wall disruption [44]. In contrast, acetonitrile is used together with formic acid in the “formic acid extraction” procedure to improve cell wall disruption [45]. Of the 30 isolates, 23 (76.7%) obtained a score of ≥2.00, indicating an identification at species level (Table S1). Overall, MALDI-TOF MS with the Bruker Biotyper identified 61 (89.7%) LAB isolates at species level. For the remaining seven isolates, MALDI-TOF scores in the range of 1.70–1.99 were obtained (Table S1) based on a comparison with the producer’s reference database. Andersen et al. (2014) even considered MALDI-TOF (Bruker Biotyper) scores in this range as acceptable for identification of *Lactobacillus* species [30]. Accordingly, six remaining isolates could be assigned to the species *Lb. acidipiscis*. As previously reported [35], the extension of the reference database could probably improve the performance of MALDI-TOF MS with the Bruker Biotyper for the classification of *Lb. acidipiscis* and *E. viikkiensis* isolates, which had the lowest average scores in this study. For example, two *Lb. futsaii* isolates were originally identified as *Lb. farciminis* with an average score of 1.88. After establishing an in-house database using the two *Lb. futsaii* reference strains CS3 and CS5 [46], they were identified as *Lb. futsaii* with an average log(score) of 2.28 (Table S1). 

### 3.2. Fingerprinting and Typing of LAB by (GTG)_5_-PCR

The 68 LAB isolates were subjected to (GTG)_5_-PCR fingerprinting technique for genotypic grouping. The dendrogram of all obtained (GTG)_5_-PCR patterns is shown in Figure 1. Setting a cut-off value at 63% similarity, the 68 isolates were grouped into seven separate clusters (I, II, IV–VIII) and one singleton (III; Figure 1). According to the results of 16S rDNA sequencing and MALDI-TOF MS, each cluster is well-differentiated and represents an individual species [*Lb. acidipiscis* (I), *Lb. fermentum* (II), *Lb. plantarum* (IV), *E. faecium* (V), *Lb. futsaii* (VI), *Lb. namurensis* (VII), and *P. pentosaceus* (VIII)]. The only singleton is an *Enterococcus* strain (III) either belonging to the species *E. viikkiensis* (16S rDNA sequencing) or *E. hermaniensis* (MALDI-TOF MS). Both species are closely related [26]. As previously reported, these results demonstrate that (GTG)_5_-PCR fingerprinting is a useful tool for grouping lactobacilli [20,47,48]. 

A high concordance of 95.6% (65/68) was assessed between 16S rDNA sequencing and MALDI-TOF MS with the Bruker Biotyper. However, one isolate (41e) was identified as *Lb. sucicola* by 16S rDNA sequencing and as *Lb. acidipiscis* by MALDI-TOF MS. Interestingly, this isolate was displayed in the *Lb. acidipiscis* cluster by (GTG)_5_-PCR fingerprinting (Figure 1). According to the literature both species are members of the phylogenetic *Lb. salivarius* group [49]. Furthermore, this data supports the finding of Dušková et al. (2012), who proved that MALDI-TOF MS with Bruker Biotyper is superior in the identification of lactobacilli species [35]. Similarly, the *Lb. plantarum/Lb. paraplantarum/Lb. pentosus* cluster determined by 16S rDNA sequencing could be divided into one *Lb. plantarum* and two *Lb. pentosus* sub-clusters based on the dendrogram and MALDI-TOF MS (Figure 1). Next to these three phylogenetically similar sub-clusters, a *Lb. zymae* strain was also assigned to this cluster. This species was recently transferred from the *Lb. buchneri* clade to the *Lb. brevis* clade [50], which is close to the *Lb. plantarum* clade [51,52]. 

Only strains with different fingerprints (e.g., 41b, 41d) or the same fingerprint and different origins (e.g., 44d, 45a) were considered for further evaluations. Isolates with a 100% similarity and the same origin were regarded as multiple isolates representing a single strain (e.g., 34d-B, 34d-S, 34b-B, 34b-S). Choosing only one of these multiple isolates (e.g., 34b-S), the initial 68 LAB isolates were reduced to 50 strains by (GTG)_5_-PCR fingerprinting technique. These 50 strains were used for GABA screening and quantification. For simplicity, only species names determined by MALDI-TOF MS were furthermore applied.

### 3.3. Prevalence of LAB in Cambodian Fermented Foods

Strains of *Lb. acidipiscis* were only found in Cambodian fermented fish (*paork chav* and *mam trey*). The species *Lb. fermentum*, *Lb. plantarum*, *Lb. pentosus*, *Lb. namurensis*, *Lb. futsaii*, and *Lb. zymae* were detected in *paork kampeus*, *mam lahong*, and *spey chrourk*, fermented foods mainly made of tiny freshwater shrimp, green papaya, and mustard (Table 1, Table 2). 

These findings are in agreement with previous studies [18,39,53,54,55]. Thus, Lb. acidipiscis was originally determined in fermented fish [53]. In general, LAB are identified as important components of the gut microbiota of fish. Members of the Lactobacillus, Lactococcus, Leuconostoc, Enterococcus, Carnobacterium, Pediococcus, Streptococcus and Weissella genera have already been isolated including Lb. fermentum, Lb. plantarum, E. durans, E. faecalis, E. pseudoavium, E. faecium, and P. pentosaceus [56,57]. Besides fermented fish, Lb. fermentum and Lb. plantarum were also found in fermented mustard and onion [14,18,39,54,55]. Followed by Lb. plantarum, Lb. pentosus was the predominant species in fermented olives [58]. Also Lb. namurensis and Lb. zymae seem to be plant-associated as these species were detected in sourdough [59]. In this context, it is assumed that LAB come from flour and may originate from wheat [60]. Furthermore, Lb. namurensis was present in fermented rice bran [61] and Lb. zymae in kimchi [62] and fermented onion [54]. In this study, Lb. zymae and Lb. namurensis were detected in fermented green papaya (mam lahong), whereas Lb. namurensis was additionally found in fermented mustard (spey chrourk) (Table 2). Likewise, Lb. futsaii was identified in fermented mustard, which corresponds to the literature [63]. It is well known that LAB represent a subdominant part of the microbiota of raw vegetables and fruits [60]. Table 2 shows that P. pentosaceus strains were present in fermented fish (prahok and trey proheum) and shrimp products (kapi and paork kampeus). This species was already isolated from fermented vegetables [18], fermented fish [55,57], and seafoods [64,65]. Next to P. pentosaceus, enterococci were detected in salted fish (Table 2). While P. pentosaceus and E. faecium can grow in the presence of ≥6.5% NaCl [66,67], salt resistance is strain-dependent in the species E. hermanniensis [68]. As enterococci are found in a variety of different ecological environments including surface and waste waters, their association with fermented seafood [64,69,70] can be explained.

### 3.4. Screening LAB Strains for GABA Production and GABA Quantification

All LAB strains were screened for their potential to produce GABA on TLC silica plates (Figure S1). Only strains with a Rf value corresponding to that of the GABA standard (0.27 cm) were selected for GABA quantification. Compared to this value, just six strains (12%) are GABA producers (Figure S1). These strains belong to the species (number of strains) *Lb. plantarum* (three), *Lb. namurensis* (two), and *Lb. futsaii* (one). Previous studies also reported mainly strains of the genus *Lactobacillus* as GABA-producing LAB [10]. Thus, *Lb. plantarum* strains from kimchi [71], cheese [72] and other traditional fermented food products [14] have already been recognized to produce GABA. In addition, *Lb. namurensis* and *Lb. futsaii* strains, isolated from Thai fermented pork sausages and shrimp products, have been identified as GABA-producing LAB [22,73,74]. According to the literature [62,75], *Lb. fermentum* and *Lb. zymae* strains have also been indicated as GABA producers, but within this work no GABA production could be determined for these *Lactobacillus* species as well as for *Lb. acidipiscis* and *Lb. pentosus*. Some studies also described GABA-producing *P. pentosaceus* strains, which were found in various fermented foods such as fermented beef or pork and alpine cheeses [6,73,74,76]. There have been only few studies on GABA-producing enterococci from fermented foods. This might be due to lower GABA production levels of *Enterococcus* strains compared to those of the genus *Lactobacillus*. Therefore, a GABA production of 1.56 mM by an *E. faecium* strain from Korean traditional fermented food was considered as high-level GABA production [77]. Nevertheless, single *E. durans* and *E. avium* strains with higher GABA yields were reported from Italian cheese and Korean fermented seafood [72,78]. In this study, however, no GABA production could be verified for *P. pentosaceus* or *Enterococcus* strains. 

The two *Lb. plantarum* strains 45a and 44d showed stronger spots for GABA production on TLC silica plates than the other four strains (Figure S1). These strains were isolated from *paork kampeus*. Thus, they may originate from tiny freshwater shrimp, but roasted rice, green papaya and galangal could also be their sources, as they are all main ingredients of *poark kampeus*. The origin of the *Lb. plantarum* strain 37e with a weaker spot is probably green papaya. Similar to *paork kampeus*, however, it can also be tiny fermented fish, roasted rice or galangal because these are components of *mam lahong* as well (Table 1). When incubated at 30 °C for 48 h in MRS broth supplemented with MSG (2%, *w*/*v*), the two *Lb. plantarum* strains 45a and 44d also produced higher concentrations of GABA (20.34 ± 1.41 mM and 16.47 ± 1.91 mM, respectively), whereas the *Lb. plantarum* strain 37e was only able to produce 5.63 ± 0.68 mM GABA (Table 3). Even lower GABA concentrations were obtained from the *Lb. futsaii* (4.68 ± 0.87 mM) and the two *Lb. namurensis* (1.62 ± 0.43 mM and 1.19 ± 0.66 mM, respectively) strains, which were isolated from fermented green papaya and mustard (Table 1). 

To our best knowledge, the highest GABA-producing *Lb. plantarum* strain isolated from fermented food produced 30.54 mM GABA (3.15 g/kg) [79]. Furthermore, one *Lb. namurensis* and two *Lb. futsaii* strains from Thai fermented shrimp and Thai fermented sausages synthesizing higher GABA concentrations [71.18 mM (7.34 g/L) and >77.58 mM (>8.00 g/L), respectively] were previously described [46,74]. However, such comparisons should be treated with caution as results from different studies are received under various conditions. Thus, the food source of the strain might have an influence on the GABA production level. In this respect, it was found that acidic food could be the habitat of high GABA producers as these can maintain the intracellular pH under acidic conditions by eliminating intracellular protons during the decarboxylation of glutamate [80]. Of course, GABA-rich foods themselves can also be a good origin of high GABA producing LAB. In addition to a wide range of traditional fermented foods such as yogurt, cheese, kimchi, sourdough and paocai [10], substantial amounts of GABA were also found in germinated edible seeds and sprouts as well as in tomato during the mature green stage [81]. However, it is believed that the accumulation of GABA in these foods is due to the presence and activity of enzymes and not to microorganisms [82]. Moreover, LAB are often selectively isolated from food based on their capacity to form high levels of GABA by supplementing culture media with MSG [46]. Also, the applied detection method (e.g. enzyme assay, chromatography or automatic amino acid analyzer) has an effect on the determination of GABA level [83,84]. It was noted that the GABA levels determined by high-performance liquid chromatography (HPLC) were lower than those measured by the GABase assay [85]. According to the authors, media with different amounts of MSG as well as the extraction and derivatisation processes required for HPLC might have been the reason for this difference [85]. Finally, the production of GABA itself can be affected and optimized by different factors, of which the most common and essential ones are pH, temperature, the ingredients and additives in the media, as well as the fermentation time [10]. For example, the GABA production of the aforementioned *E. faecium* strain was increased from 1.56 mM to 14.86 mM when this strain was cultivated in a specially designed medium under optimal conditions [77]. These requirements vary among microorganisms due to the different properties of their GADs [10]. Hence, the recommended step for optimizing GABA production is the characterization of these properties in the relevant *Lactobacillus* strains and the development of efficient production processes. When fermenting glutamate-rich foods with well-characterized starter cultures and the addition of exogenous MSG, GABA-concentrations of approximately 101.82 mM (10.5 g/kg) could be finally achieved [86].

## 4. Conclusions

A total of 68 LAB isolates from different Cambodian fermented foods were identified by genotypic and proteomic techniques. Applying rep-PCR (GTG)_5_, the initial number of LAB was reduced to 50 strains, which were screened for GABA production. Six strains belonging to the species *Lb. plantarum*, *Lb. futsaii*, and *Lb. namurensis* were able to produce GABA, in particular one *Lb. plantarum* strain showed the highest GABA concentration (20.34 mM), followed by another *Lb. plantarum* strain (16.47 mM). Since GABA is used as an active component in foods and pharmaceuticals, these GABA-producing strains could be of interest for the production of GABA-enriched fermented foods and beverages. However, in order to further increase and optimize the GABA production, detailed characterization of these strains is needed, including research on their safety, technological performance and other probiotic attributes. Such starter cultures would allow the production of Cambodian fermented foods with an additional nutritional value that meet the population’s dietary habits and are also more attractive for the international food market. 

## Figures and Tables

**Table 1:** Selected Cambodian fermented food samples.

No.	Local Name (Number of Samples)	English Name	Ingredients	Usage	Market Origin
1	*Prahok* (*n* = 1)	Fish paste	Freshwater fish, salt	Main dish, side-dish, condiment, seasoning	Chamkadaung
2	*Paork chav* (*n* = 3)	Fermented fish	Freshwater fish, brown glutinous rice, salt	Main dish, side-dish	Oreusey
3	*Mam trey* (*n* = 1)	Fermented fish	Freshwater fish, palm sugar, salt	Main dish, side-dish	Thmey
4	*Trey proheum* (*n* = 2)	Salted fish	Freshwater fish, salt	Main dish, seasoning	Thmey
5	*Kapi* (*n* = 1)	Shrimp paste	Tiny marine shrimp, salt	Side-dish, condiment, seasoning	Chas
6	*Paork kampeus* (*n* = 3)	Fermented tiny freshwater shrimp	Tiny freshwater shrimp, salt, roasted rice, slightly green papaya, galangal	Side-dish	Phumreusey, Limcheanghak
7	*Mam lahong* (*n* = 2)	Fermented green papaya	Green papaya, slightly tiny fermented fish, salt, roasted rice, galangal	Side-dish	Limcheanghak
8	*Spey chrourk* (*n* = 2)	Fermented mustard	Chinese mustard, salt	Side-dish	Phumreusey, Limcheanghak

**Table 2:** Identification of LAB from different fermented foods by partial 16S rDNA sequencing and MALDI-TOF MS.

Food Samples	Total Number of Isolates	Identification Technique			
		Partial 16S rDNA	Number of Identified Isolates	MALDI-TOF MS (Bruker Biotyper)	Number of Identified Isolates
*Prahok* (*n* = 1)	1	*P. pentosaceus*	1	*P. pentosaceus*	1
*Paork chav* (*n* = 3)	13	*Lb. acidipiscis* *Lb. sucicola*	12 1	*Lb. acidipiscis* –	13 0
*Mam trey* (*n* = 1)	5	*Lb. acidipiscis*	5	*Lb. acidipiscis*	5
*Trey proheum* (*n* = 2)	7	*P. pentosaceus* *E. faecium* – *E. viikkiensis/E. durans/E. malodoratus/E. pseudoavium* *E. pseudoavium/E. avium*	3 2 0 1 1	*P. pentosaceus* *E. faecium* *E. hermanniensis* – –	3 3 1 0 0
*Kapi* (*n* = 1)	1	*P. pentosaceus*	1	*P. pentosaceus*	1
*Paork kampeus* (*n* = 3)	16	*Lb. fermentum* *Lb. plantarum/Lb. paraplantarum/Lb. pentosus* *P. pentosaceus*	9 4 3	*Lb. fermentum* *Lb. pentosus* *Lb. plantarum* *P. pentosaceus*	9 2 2 3
*Mam lahong* (*n* = 2)	12	*Lb. plantarum/Lb. paraplantarum/Lb. pentosus* *Lb. namurensis* *Lb. zymae*	8 3 1	*Lb. pentosus* *Lb. plantarum* *Lb. namurensis* *Lb. zymae*	6 2 3 1
*Spey chrourk* (*n* = 2)	13	*Lb. fermentum* *Lb. plantarum/Lb. paraplantarum/Lb. pentosus* *Lb. namurensis* *Lb. futsaii*	9 1 1 2	*Lb. fermentum* *Lb. pentosus* *Lb. namurensis* *Lb. futsaii*	9 1 1 2

(–) species that was not identified by the respective technique.

**Table 3:** LAB with GABA-producing abilities after 48 h cultivation.

Strain	LAB Species Identified by MALDI-TOF MS (Bruker Biotyper)	Corresponding Lanes of TLC Analysis	Rf (cm) ^a^	GABA (mM) ^b^ Production
45a	*Lb. plantarum*	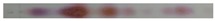	0.27	20.34 ± 1.41
44d	*Lb. plantarum*	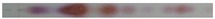	0.27	16.47 ± 1.91
37e	*Lb. plantarum*	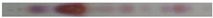	0.27	5.63 ± 0.68
32d	*Lb. futsaii*	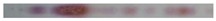	0.27	4.68 ± 0.87
37b	*Lb. namurensis*	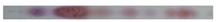	0.27	1.62 ± 0.43
32c	*Lb. namurensis*	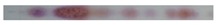	0.27	1.19 ± 0.66
-	GABA-negative strain ^c^	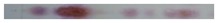	0.16	n.d.
-	GABA standard	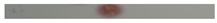	0.27	n.d.
-	MSG ^d^	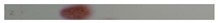	0.16	n.d.

^a^ Rf = retention factor, defined as the ratio of the distance traveled by the center of a spot to the distance traveled by the solvent front; only strains showing the same Rf value as the GABA standard (=0.27 cm) were selected for GABA (mM) quantification. ^b^ mean ± SD. ^c^ GABA-negative strain = any strain that does not produce a GABA spot with a Rf value equal to that of the GABA standard (e.g. all tested strains except of 45a, 44d, 37e, 32d, 37 b, 32c). ^d^ MSG = monosodium glutamate. n.d.= not determined.

## Supplementary Materials

The following are available online at https://www.mdpi.com/2218-273X/9/12/768/s1, Figure S1: Thin-layer chromatography (TLC) analysis of GABA producing-LAB, Table S1: List of LAB isolates identified by partial 16S rDNA sequencing (% similarity, accession number) and MALDI-TOF MS (Bruker Biotyper) log(score) and their source.
