# Supplementary material for: Identification, Classification and Screening for γ-Amino-butyric Acid Production in Lactic Acid Bacteria from Cambodian Fermented Foods
Source: Biomolecules. 2019 Nov 22;9(12):768. doi: 10.3390/biom9120768 (PMC6995518; doi:10.3390/biom9120768)
Supplement: Supplementary file 1 [file biomolecules-09-00768-s001.pdf]

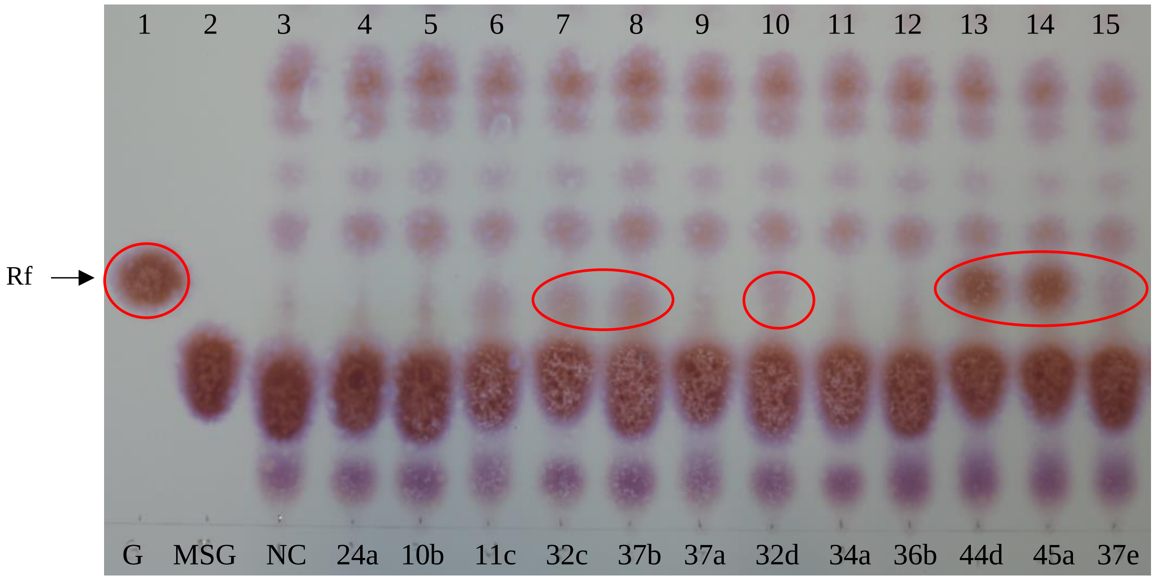

Figure S1. Thin-layer chromatography (TLC) analysis of GABA producing-LAB. Lane 1: GABA standard (G); lane 2: monosodium glutamate (MSG); lane 3: negative control (NC); lane 7–8, 10, 13–15: GABA-positive strains (*Lb. namurensis*, *Lb. futsaii*, *Lb. plantarum*); lane 4–6, 9, 11–12: GABA-negative strains (any strain that does not produce a GABA spot with a Rf value equal to that of the GABA standard (e.g. all tested strains except of 45a, 44d, 37e, 32d, 37 b, 32c).

Table S1: List of LAB isolates identified by partial 16S rDNA sequencing (% similarity, accession number) and MALDI-TOF MS (Bruker Biotyper) log(score) and their source

Isolates	16S rDNA sequencing<br>Closest relative(s) (% similarity, accession n.)	MALDI-TOF MS<br>Best hit organism based on log(score)	Log(score)	Source
32e-S	*Lb. fermentum* (99, NR_104927.1)	*Lb. fermentum*	2.20[a]	*Spey chrourk*
32e-B	*Lb. fermentum* (99, NR_104927.1)	*Lb. fermentum*	2.20[a]	–
34a	*Lb. fermentum* (100, NR_104927.1)	*Lb. fermentum*	2.20[b]	–
34b-S	*Lb. fermentum* (99, NR_104927.1)	*Lb. fermentum*	2.02[b]	–
34b-B	*Lb. fermentum* (100, NR_104927.1)	*Lb. fermentum*	2.12[b]	–
34c	*Lb. fermentum* (99, NR_104927.1)	*Lb. fermentum*	2.00[b]	–
34d-S	*Lb. fermentum* (99, NR_104927.1)	*Lb. fermentum*	2.02[b]	–
34d-B	*Lb. fermentum* (99, NR_104927.1)	*Lb. fermentum*	2.02[b]	–
34e	*Lb. fermentum* (99, NR_104927.1)	*Lb. fermentum*	2.12[b]	–
44a	*Lb. fermentum* (99, NR_104927.1)	*Lb. fermentum*	2.00[b]	*Paork kampeus*
44b	*Lb. fermentum* (100, NR_104927.1)	*Lb. fermentum*	2.05[b]	–
44e	*Lb. fermentum* (100, NR_104927.1)	*Lb. fermentum*	2.07[b]	–
45b	*Lb. fermentum* (99, NR_104927.1)	*Lb. fermentum*	2.09[b]	–
45c	*Lb. fermentum* (100, NR_104927.1)	*Lb. fermentum*	2.02[a]	–
45d	*Lb. fermentum* (100, NR_104927.1)	*Lb. fermentum*	2.08[b]	–
46a	*Lb. fermentum* (100, NR_104927.1)	*Lb. fermentum*	2.09[b]	–
46e-S	*Lb. fermentum* (99, NR_104927.1)	*Lb. fermentum*	2.18[b]	–
46e-B	*Lb. fermentum* (100, NR_104927.1)	*Lb. fermentum*	2.15[a]	–
22a	*Lb. acidipiscis* (99, NR_112693.1)	*Lb. acidipiscis*	2.07[b]	*Mam trey*
22b	*Lb. acidipiscis* (99, NR_112693.1)	*Lb. acidipiscis*	2.09[b]	–
22c	*Lb. acidipiscis* (99, NR_112693.1)	*Lb. acidipiscis*	2.04[b]	–
22d	*Lb. acidipiscis* (99, NR_112693.1)	*Lb. acidipiscis*	2.11[b]	–
22e	*Lb. acidipiscis* (99, NR_112693.1)	*Lb. acidipiscis*	2.03[b]	–
42a	*Lb. acidipiscis* (98, NR_112693.1)	*Lb. acidipiscis*	1.79[b,c]	*Paork chav*
42b	*Lb. acidipiscis* (99, NR_112693.1)	*Lb. acidipiscis*	1.97[b,c]	–
42c	*Lb. acidipiscis* (99, NR_112693.1)	*Lb. acidipiscis*	2.00[b]	–
42d	*Lb. acidipiscis* (99, NR_112693.1)	*Lb. acidipiscis*	1.87[b,c]	–
42e	*Lb. acidipiscis* (99, NR_112693.1)	*Lb. acidipiscis*	2.00[b]	–
43a	*Lb. acidipiscis* (99, NR_112693.1)	*Lb. acidipiscis*	1.80[b,c]	–
43b	*Lb. acidipiscis* (99, NR_112693.1)	*Lb. acidipiscis*	2.04[b]	–
43c	*Lb. acidipiscis* (99, NR_112693.1)	*Lb. acidipiscis*	1.95[b,c]	–
41a	*Lb. acidipiscis* (99, NR_112693.1)	*Lb. acidipiscis*	1.86[b,c]	–
41b	*Lb. acidipiscis* (99, NR_112693.1)	*Lb. acidipiscis*	2.01[b]	–
41c	*Lb. acidipiscis* (99, NR_112693.1)	*Lb. acidipiscis*	2.32[a]	–
41d	*Lb. acidipiscis* (99, NR_112693.1)	*Lb. acidipiscis*	2.11[a]	–
41e	*Lb. sucicola* (98, NR_112785.1)	*Lb. acidipiscis*	2.25[a]	–
32a	*Lb. futsaii* (99, NR_117973.1)	*Lb. futsaii*	2.26[a]	*Spey chrourk*
32d	*Lb. futsaii* (99, NR_117973.1)	*Lb. futsaii*	2.30[a]	–
32c	*Lb. namurensis* (99, NR_042514.1)	*Lb. namurensis*	2.43[a]	–
36d	*Lb. namurensis* (99, NR_042514.1)	*Lb. namurensis*	2.54[a]	*Mam lahong*
36e	*Lb. namurensis* (99, NR_042514.1)	*Lb. namurensis*	2.24[a]	–
37b	*Lb. namurensis* (99, NR_042514.1)	*Lb. namurensis*	2.45[a]	–
37a	*Lb. zymae* (97, NR_042241.1)	*Lb. zymae*	2.04[a]	–
36a-S	*Lb. plantarum* (99, NR_104573.1)<br>*Lb. paraplantarum* (99, NR_025447.1)<br>*Lb. pentosus* (99, NR_029133.1)	*Lb. pentosus*	2.38[a]	–
36a-B	*Lb. plantarum* (99, NR_104573.1)<br>*Lb. paraplantarum* (99, NR_025447.1)<br>*Lb. pentosus* (99, NR_029133.1)	*Lb. pentosus*	2.28[a]	–
36b-S	*Lb. plantarum* (99, NR_104573.1)	*Lb. pentosus*	2.41[a]	–

	Lb. paraplantarum (99, NR_025447.1)			
	Lb. pentosus (99, NR_029133.1)			
36b-B	*Lb. plantarum* (99, NR_104573.1)	*Lb. pentosus*	2.40[a]	–
	Lb. paraplantarum (99, NR_025447.1)			
	Lb. pentosus (99, NR_029133.1)			
36c	*Lb. plantarum* (99, NR_104573.1)	*Lb. pentosus*	2.20[a]	–
	Lb. paraplantarum (99, NR_025447.1)			
	Lb. pentosus (99, NR_029133.1)			
36f	*Lb. plantarum* (99, NR_104573.1)	*Lb. pentosus*	2.26[a]	–
	Lb. paraplantarum (99, NR_025447.1)			
	Lb. pentosus (99, NR_029133.1)			
32b	*Lb. plantarum* (99, NR_104573.1)	*Lb. pentosus*	2.28[a]	*Spey chrourk*
	Lb. paraplantarum (99, NR_025447.1)			
	Lb. pentosus (99, NR_029133.1)			
45e	*Lb. plantarum* (99, NR_104573.1)	*Lb. pentosus*	2.33[a]	*Paork kampeus*
	Lb. paraplantarum (99, NR_025447.1)			
	Lb. pentosus (99, NR_029133.1)			
46b	*Lb. plantarum* (99, NR_104573.1)	*Lb. pentosus*	2.14[a]	–
	Lb. paraplantarum (99, NR_025447.1)			
	Lb. pentosus (99, NR_029133.1)			
44d	*Lb. plantarum* (99, NR_104573.1)	*Lb. plantarum*	2.39[a]	–
	Lb. paraplantarum (99, NR_025447.1)			
	Lb. pentosus (99, NR_029133.1)			
45a	*Lb. plantarum* (99, NR_104573.1)	*Lb. plantarum*	2.43[a]	–
	Lb. paraplantarum (99, NR_025447.1)			
	Lb. pentosus (99, NR_029133.1)			
37c	*Lb. plantarum* (99, NR_104573.1)	*Lb. plantarum*	2.34[a]	*Mam lahong*
	Lb. paraplantarum (99, NR_025447.1)			
	Lb. pentosus (99, NR_029133.1)			
37e	*Lb. plantarum* (99, NR_104573.1)	*Lb. plantarum*	2.38[a]	–
	Lb. paraplantarum (99, NR_025447.1)			
	Lb. pentosus (99, NR_029133.1)			
10b	*E. faecium* (99, NR_114742.1)	*E. faecium*	2.35[a]	*Trey proheum*
11b	*E. faecium* (99, NR_114742.1)	*E. faecium*	2.45[a]	–
11d	*E. pseudoavium* (96, NR_114785.2)	*E. faecium*	2.25[a]	–
	E. avium (96, NR_114777.1)			
11c	*E. viikkiensis* (99, NR_117976.1)	*E. hermanniensis*	1.87[b,c]	–
	E. durans (99, NR_036922.1)			
	E. malodoratus (99, NR_114453.1)			
	E. pseudoavium (99, NR_028705.1)			
10a	*P. pentosaceus* (99, NR_042058.1)	*P. pentosaceus*	2.04[a]	–
11a	*P. pentosaceus* (98, NR_042058.1)	*P. pentosaceus*	2.17[a]	–
11e	*P. pentosaceus* (100, NR_042058.1)	*P. pentosaceus*	2.09[a]	–
24a	*P. pentosaceus* (100, NR_042058.1)	*P. pentosaceus*	2.03[a]	*Kapi*
44c	*P. pentosaceus* (100, NR_042058.1)	*P. pentosaceus*	2.15[a]	*Paork kampeus*
46c	*P. pentosaceus* (100, NR_042058.1)	*P. pentosaceus*	2.04[a]	–
46d	*P. pentosaceus* (99, NR_042058.1)	*P. pentosaceus*	2.18[a]	–
51a	*P. pentosaceus* (99, NR_042058.1)	*P. pentosaceus*	2.25[a]	*Prahok*

[a]"extended direct transfer" procedure.
[b]"formic acid extraction" procedure.
[c]log(score) 1.70–<2.00: identification at genus level, log(score) ≥2.00: identification at species level.
